# Precision Gas Sensing Interface Circuit with Digital Potentiometer-Based Dynamic Gain Control

**DOI:** 10.3390/s26092887

**Published:** 2026-05-05

**Authors:** Soon-Kyu Kwon, Hyeon-June Kim

**Affiliations:** Department of Semiconductor Engineering, Seoul National University of Science and Technology, Seoul 01811, Republic of Korea; skkwon@seoultech.ac.kr

**Keywords:** digital potentiometer, analog front-end (AFE), gas sensor interface, saturation prevention

## Abstract

This paper proposes a digital potentiometer-based adaptive gas sensor interface for stable detection without signal saturation under extreme environmental fluctuations. Conventional fixed-gain circuits often suffer from limited dynamic range, leading to data loss when severe baseline drifts exceed ADC input limits. To address this, we developed a real-time control algorithm that actively adjusts attenuator and amplifier gains, maintaining the ADC input voltage (V_ADC_) near the common-mode voltage (V_CM_). Experimental results demonstrate that the interface remains stable even when the buffer voltage reaches 2.75 V, significantly surpassing the 1.2 V ADC limit. Sensor resistance data, reconstructed by inversely calculating updated circuit parameters, achieved high accuracy with a Mean Absolute Percentage Error (MAPE) of 1.628% and a maximum relative error under 4.8%. Consequently, this study proves that logically extending the physically limited ADC dynamic range enables high-precision gas sensing in diverse environments without requiring high-performance computing devices. This approach provides a cost-effective and robust solution for compact IoT-based gas monitoring systems.

## 1. Introduction

**Figure 1:**
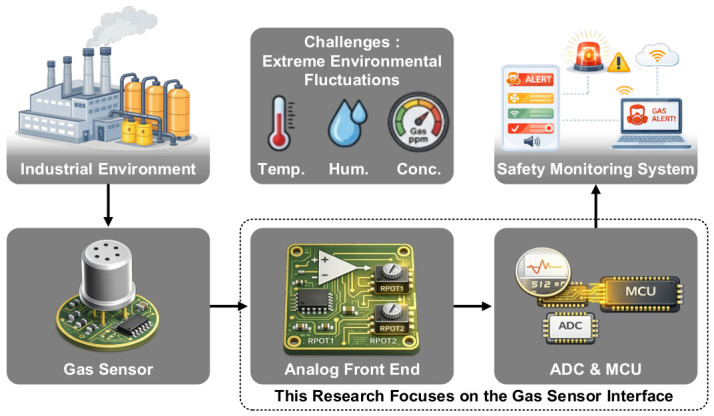
General architecture of a gas monitoring system in industrial environments.

Gas sensor systems play a pivotal role in various modern fields, including environmental monitoring, industrial safety, and scientific research [1,2]. These technologies contribute significantly to detecting toxic gas leaks and ensuring workplace safety by converting chemical gas concentrations into reliable electrical signals [3,4]. Figure 1 illustrates the architecture of a typical gas sensing system deployed in harsh industrial environments. Such systems are challenged not only with detecting target gases with ppm-level precision but also with enduring and compensating for extreme environmental fluctuations in real time, such as rapid changes in temperature and humidity that cause significant disturbances to the sensor module [5,6,7]. These environmental factors directly affect the surface reactions of semiconductor-based gas sensors, leading to severe baseline drift and output signal dispersion, which serve as primary obstacles in sensor interface design [8,9,10]. To enhance sensor stability, researchers have explored structural modifications and optimized cell designs, such as localized micro-heater integration, electrode geometry optimization, and the use of specialized catalyst layers to minimize baseline drift [11,12,13]. While these sensor-level advancements significantly improve intrinsic stability, they often involve complex fabrication processes and are specifically tailored to certain sensing materials. Moreover, even with optimized cell designs, complete elimination of environmental cross-sensitivity remains a fundamental challenge due to the inherent physicochemical properties of gas-surface interactions. Therefore, to achieve a truly comprehensive and robust sensing system, an interface-level approach that can dynamically compensate for irreducible signal fluctuations is not merely a supplementary tool but a necessary requirement to overcome these physical constraints.

Conventional fixed-gain interface circuits have a very limited dynamic range to accommodate the wide resistance variations exhibited by gas sensors [14,15]. The most critical technical challenge arising at the analog front-end (AFE), analog-to-digital converter (ADC), and microcontroller unit (MCU) stages shown in Figure 1 is signal saturation. When the voltage change caused by exposure to high humidity or high gas concentrations exceeds the allowable ADC input range, data loss occurs as the signal is clipped at the upper or lower thresholds. Conversely, if the gain is set low to prevent such saturation in conventional architectures, the LSB resolution of the ADC becomes insufficient in low-concentration regions, resulting in a performance degradation where fine concentration changes cannot be identified. In short, achieving both a wide detection range and high precision simultaneously is a difficult task for traditional static circuit structures [16].

To overcome these limitations, various adaptive AFE designs and readout systems have been investigated [17,18,19]. However, these conventional approaches often require complex analog feedback loops and multi-stage auto-scaling circuits, or they rely on high-performance digital processing utilizing complex compensation algorithms such as neural networks. These architectures inevitably demand high-specification MCUs and multiple external components, leading to increased power consumption and system footprint. While the integration of an MCU is indispensable for real-time control and data acquisition in modern adaptive systems, minimizing its required specifications is a critical design factor. By effectively offloading the dynamic range extension task to a simple digital potentiometer (DPOT)-based hardware level, our proposed architecture significantly lowers the computational burden. This enables the system to be fully driven by a low-end MCU, providing substantial benefits in terms of both manufacturing cost and overall power consumption. In this paper proposes a highly efficient adaptive gas sensor interface utilizing a DPOT to collect a wide range of sensor signals without loss, even within the constrained input range of an ADC. The proposed method employs an active feedback loop to adjust the circuit’s gain and offset in real time. Specifically, it forces the input signal to constantly track the ADC’s common-mode voltage, fundamentally preventing the hardware from reaching saturation regions. This approach enables system miniaturization and real-time calibration capabilities without the need for high-performance external computing devices or complex algorithms, offering significant advantages for implementing low-power, compact IoT gas sensor modules.

The primary contributions of this research are as follows. First, we quantitatively analyze the sensor output dispersion due to environmental fluctuations and present the practical risks of hardware saturation. Second, we propose an active AFE structure that dynamically confines signals within a narrow ADC input range using an attenuator and amplifier structure based on a DPOT, achieving high reconstruction accuracy despite its low complexity. Third, the proposed circuit and control logic were implemented as a PCB-level prototype, and the precision of data recovery in saturation regions was validated through actual gas exposure experiments. Furthermore, the proposed adaptive interface is designed as a versatile platform that can be integrated with emerging multifunctional sensing systems, such as dual-mode sensors for simultaneous gas and temperature monitoring [20]. Furthermore, the proposed interface ensures data integrity by preventing signal saturation, providing a crucial hardware foundation for accurate feature extraction in future gas identification algorithms. By preserving the complete profile of the sensor response, it enables the high-fidelity data acquisition necessary for improving the accuracy of substance identification.

The remainder of this paper is organized as follows. Section 2 analyzes the dispersion and drift issues of a single gas sensor caused by temperature and humidity changes using experimental data. Section 3 discusses the ADC saturation problem in conventional structures and provides a detailed description of the proposed concept, circuit architecture, and control logic designed to receive signals stably within a narrow ADC input range. Section 4 presents the experimental results and performance verification, and finally, Section 5 concludes the paper.

## 2. Environmental Vulnerabilities and Hardware Constraints of Gas Sensors

This section analyzes the signal characteristics of the Figaro TGS-2600 [21], a commercial semiconductor gas sensor, and discusses essential design considerations for an effective readout circuit within a constrained hardware environment. Generally, gas sensors possess unique initial baseline values for each unit and react sensitively to varying ambient conditions, leading to significant signal variations and dispersion. To investigate these phenomena, the sensor’s output voltage was analyzed across various temperature and humidity ranges. To ensure the reproducibility of these measurements, the experimental environment was strictly regulated using a specialized gas flow system. Synthetic air (80% N_2_ and 20% O_2_) was employed as the carrier gas at a constant flow rate of 500 sccm via a calibrated mass flow controller (MFC). Before each measurement, the sensors were purged with fresh air for 1000 s to establish a stable initial baseline. Environmental parameters were controlled through a custom-built regulation system: humidity was modulated using a water bubbling method, while temperature was precisely adjusted via a line-heating system applied to the gas delivery pipes. Both parameters were monitored in real time using a high-precision reference sensor to maintain feedback control.

**Figure 2:**
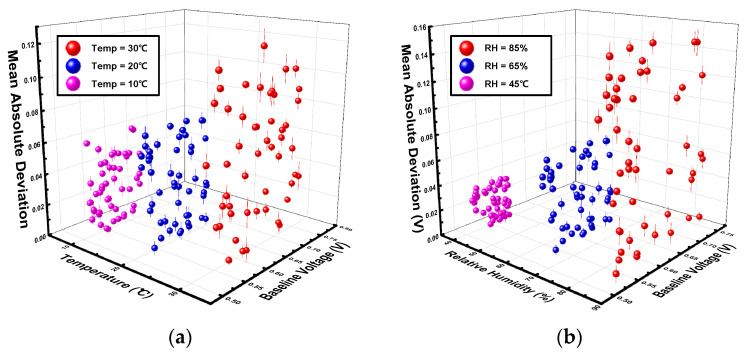
Output voltage of TGS2600 according to (**a**) temperature variations and (**b**) relative humidity changes.

Figure 2a shows the influence of temperature variations (10, 20, and 30 °C) while maintaining a constant relative humidity of 45% RH, and Figure 2b illustrates the impact of humidity changes (45, 65, and 85% RH) at a constant temperature of 20 °C. In these graphs, the *x*-axis represents the sensor’s baseline voltage, while the *z*-axis denotes the Mean Absolute Deviation (MAD) under each condition. In addition, error bars were incorporated into Figure 2 to indicate the measurement uncertainty. For each of the 80 sampled data points, five independent repeated experiments were conducted, and the corresponding standard deviations were calculated and plotted as error bars. These results confirm the high repeatability and statistical reliability of the measured sensor responses.

As shown in Figure 2a, both the baseline voltage and its deviation tended to increase as the temperature rose. This shift was confirmed to occur continuously and linearly, providing the essential design constraints for the adaptive gain control algorithm to maintain signal integrity. For instance, at 10 °C, the average baseline was 0.56 V with a maximum deviation of 0.06 V. However, at 30 °C, the average baseline rose to 0.65 V and the maximum deviation reached 0.12 V, indicating a twofold increase in signal uncertainty. Similarly, a critical trend regarding humidity variations was observed in Figure 2b. As the relative humidity increased from 45% to 85% RH, the average baseline voltage rose moderately from 0.55 V to 0.59 V, whereas the MAD surged fivefold, from 0.03 V to 0.15 V. This rapid increase in deviation at 85% RH suggests that the adsorption of water molecules in high-humidity environments severely undermines the stability of the sensor surface [22,23].

**Figure 3:**
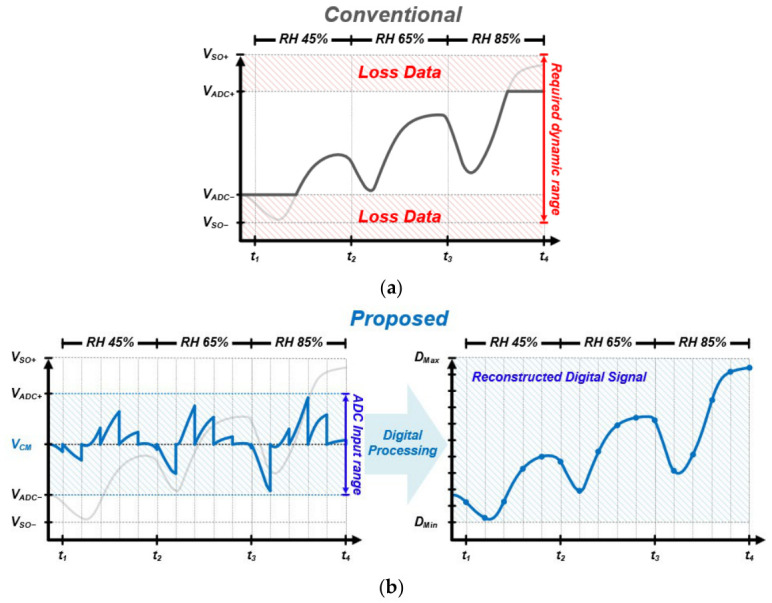
Signal processing in (**a**) conventional interface vs. (**b**) proposed adaptive interface.

Baseline shifts and the exacerbation of signal dispersion due to environmental fluctuations present significant challenges in securing the dynamic range of an Analog-to-Digital Converter (ADC) during ROIC design. Figure 3 compares the limitations of the conventional method with the proposed adaptive signal processing concept of this study. In the conventional approach, if the sensor’s output range exceeds the ADC’s allowable input limits, signal saturation occurs as shown in Figure 3a. Specifically, when the baseline rises due to environmental factors, the peak signal from the gas response reaches and is clipped at the upper threshold (V_ADC+_), resulting in the loss of critical data during high-concentration gas exposure. To address this, we propose an adaptive offset control method, as illustrated in Figure 3b. Before the signal drifts out of the ADC’s effective range, the input signal is shifted in real time toward the V_CM_ using digital potentiometers. The resulting discontinuous analog waveforms are subsequently processed through a digital reconstruction stage to recover their original linearity, eventually being transformed into a Reconstructed Digital Signal. This mechanism overcomes the physical limitations of a fixed ADC input range, ensuring that the entire sensor signal is captured without loss, even amidst extensive baseline shifts. Consequently, the proposed adaptive ROIC maximizes the system’s sensing capability by securing the maximum effective signal within a limited dynamic range.

## 3. Proposed Adaptive Gas Sensor Interface

As analyzed in Section 2, active signal conditioning is essential to accommodate the wide range of TGS-2600 sensor variations caused by environmental fluctuations within the 1.2 V ADC range. This section proposes the hardware architecture and a real-time adaptive algorithm to implement this requirement.

**Figure 4:**
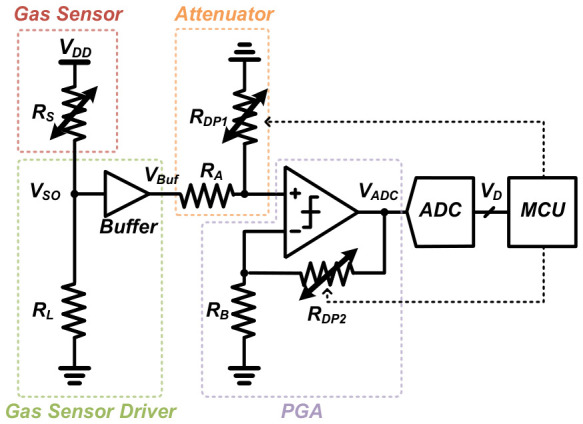
Proposed adaptive AFE with DPOT-based offset and gain control.

The overall AFE structure of the proposed adaptive gas sensor interface consists of an organic combination of a gas sensor driver, an attenuator, and a Programmable Gain Amplifier (PGA), as shown in Figure 4. First, in the gas sensor driver, a commercial gas sensor (R_S_) and a load resistor (R_L_) are connected in series to convert the resistance change from the gas response into a voltage signal, V_SO_. This signal is then buffered to output V_Buf_, ensuring impedance matching with the subsequent stages.

The signal is then delivered to the attenuator stage, composed of resistor R_A_ and the first digital potentiometer (R_DP1_). This structure functions as a voltage divider; when the baseline voltage becomes abnormally high due to environmental factors, the MCU decreases the resistance of R_DP1_ to attenuate the voltage level before it enters the amplifier. This serves as a primary defense mechanism by downward adjusting the overall offset to prevent hardware saturation. In the final stage, the PGA region features resistor R_B_ and a second digital potentiometer (R_DP2_), which form a feedback loop for a non-inverting amplifier to generate the final output voltage (V_ADC_). The MCU updates the R_DP2_ value in real time to precisely control the amplification, helping the sensor signal maintain optimal resolution within the ADC’s input range. The final ADC input voltage, V_ADC_, is determined by the transfer function combining the attenuation ratio and amplification gain:

(1) $$V_{ADC}=(V_{Buf}\times \frac{R_{DP1}}{R_{A}+R_{DP1}})\times (1+\frac{R_{DP2}}{R_{B}})$$

**Figure 5:**
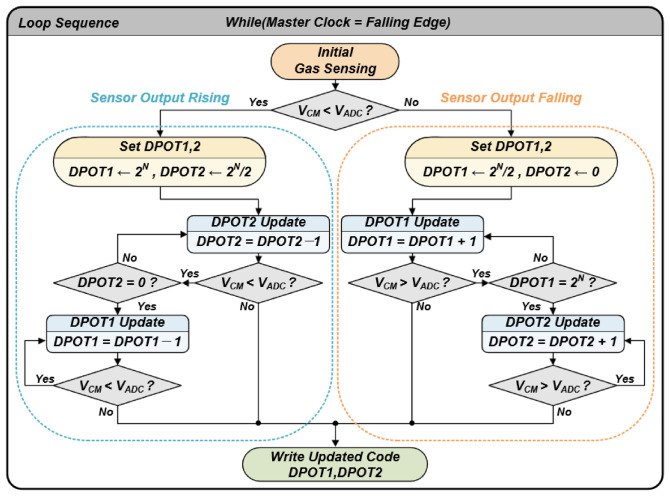
Flowchart of the adaptive algorithm.

The algorithm designed for active control of the proposed circuit features a feedback loop structure that continuously monitors signal directionality and threshold crossings, as shown in Figure 5. Synchronized with the falling edge of the master clock, the system reads the current ADC input value (V_ADC_) and compares it with the target V_CM_ to determine whether the sensor output is rising or falling. This state determination serves as a critical indicator for the direction of subsequent DPOT updates.

When a rising signal exceeds the predefined upper threshold, the algorithm first gradually decreases the R_DP2_ value to reduce the amplifier gain. If the signal remains above the reference voltage even after the gain is minimized (D_DP2_ = 0), the system decreases the R_DP1_ value in the attenuator stage to downward-adjust the input offset itself. Through this stepwise adjustment, the signal is forcibly relocated back within the ADC’s effective input range. Conversely, if a falling signal drops below the lower threshold, the system first raises the base voltage of the signal by increasing R_DP1_. Subsequently, if necessary, R_DP2_ is adjusted to stabilize the signal back within the V_CM_ region. This real-time adaptive control physically implements the sawtooth-shaped waveforms presented in Figure 3, providing an environment where large-scale sensor response data can be collected without loss, even within the narrow 1.2 V voltage range.

The most significant advantage of this adaptive algorithm lies in its ability to perfectly track the signal variations (ΔV) occurring within each control cycle. Every cycle, the system records not only the voltage values received through the ADC but also the code variations in R_DP1_ and R_DP2_ updated by the MCU. The core of this process is the mathematical reconstruction of the original buffer voltage from the hardware-adjusted V_ADC_. By inverting the signal transformation defined in Equation (1), the input signal can be precisely recovered as follows:

(2) $$V_{Buf}=(\frac{V_{ADC}}{\left(\frac{R_{DP1}}{R_{A}+R_{DP1}})\times (1+\frac{R_{DP2}}{R_{B}}\right)})$$

Equation (2) proves that the original gas response data can be seamlessly restored without information loss, even when the hardware dynamically adjusts the offset and gain to prevent saturation. Since each DPOT code corresponds directly to a physical resistance value, the original input signal information can be reconstructed by inversely calculating and summing the variations in each step, even for saw-tooth-shaped signals. Consequently, this method effectively results in the logical extension of the physically fixed 1.2 V ADC input range. Even for high-voltage signals that would have been lost due to saturation in conventional architectures, whether caused by environmental fluctuations or rapid gas responses, the proposed algorithm brings them down into the effective range for digitization, providing accurate gas concentration data across the entire dynamic range. This ensures data continuity and reliability even in extreme measurement environments that exceed the system’s physical limits.

## 4. Measurement Results and Discussion

This section provides a detailed description of the prototype hardware and the experimental setup established to verify the performance of the adaptive gas sensor interface proposed in Section 3. Furthermore, we analyze the operational waveforms of the implemented adaptive algorithm through actual gas exposure experiments. Finally, the accuracy of the proposed interface is quantitatively demonstrated by comparing the error rate between the sensor resistance values reconstructed via inverse calculation in the MCU and the actual measured sensor resistance values.

**Figure 6:**
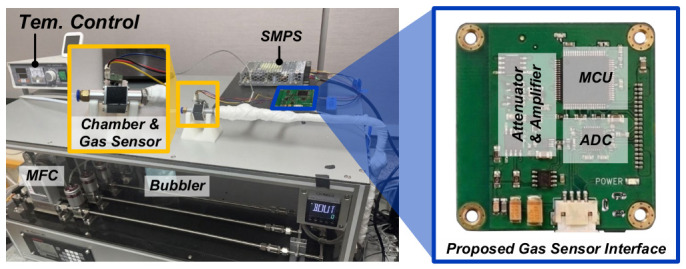
Prototype and experimental setup.

Figure 6 shows the small module and experimental setup produced to verify the performance of the proposed adaptive gas sensor interface and demonstrate the overall experimental configuration. The right side shows the prototype of the small gas sensor module implemented in this study. This module is based on the Figaro TGS2600 gas sensor and integrates an 8-bit MCU with the proposed adaptive algorithm [24], an ADC with 12-bit resolution [25], and an attenuator and amplifier circuit for signal processing on a small PCB. The experimental system shown on the left consists of an MFC system, a temperature and humidity control system, and a sensor chamber to precisely simulate gas concentration and environmental conditions. By adjusting the mixing ratio of the target gas (ethanol) and clean air for dilution in real time through MFC, it was possible to stably supply gas at the desired concentration. Additionally, temperature and humidity sensors were placed to evaluate the sensor’s environmental dependence, and various environmental conditions were implemented using a bubbler and heater connected to an external controller.

**Figure 7:**
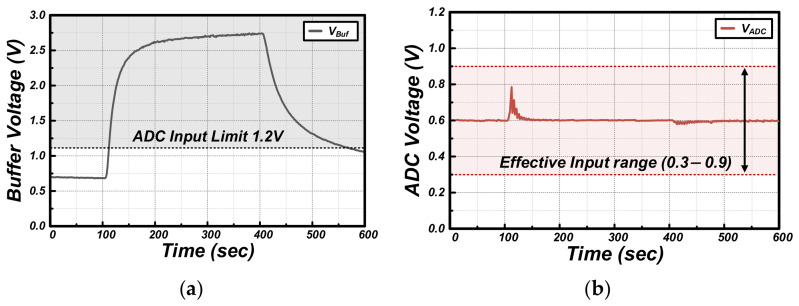

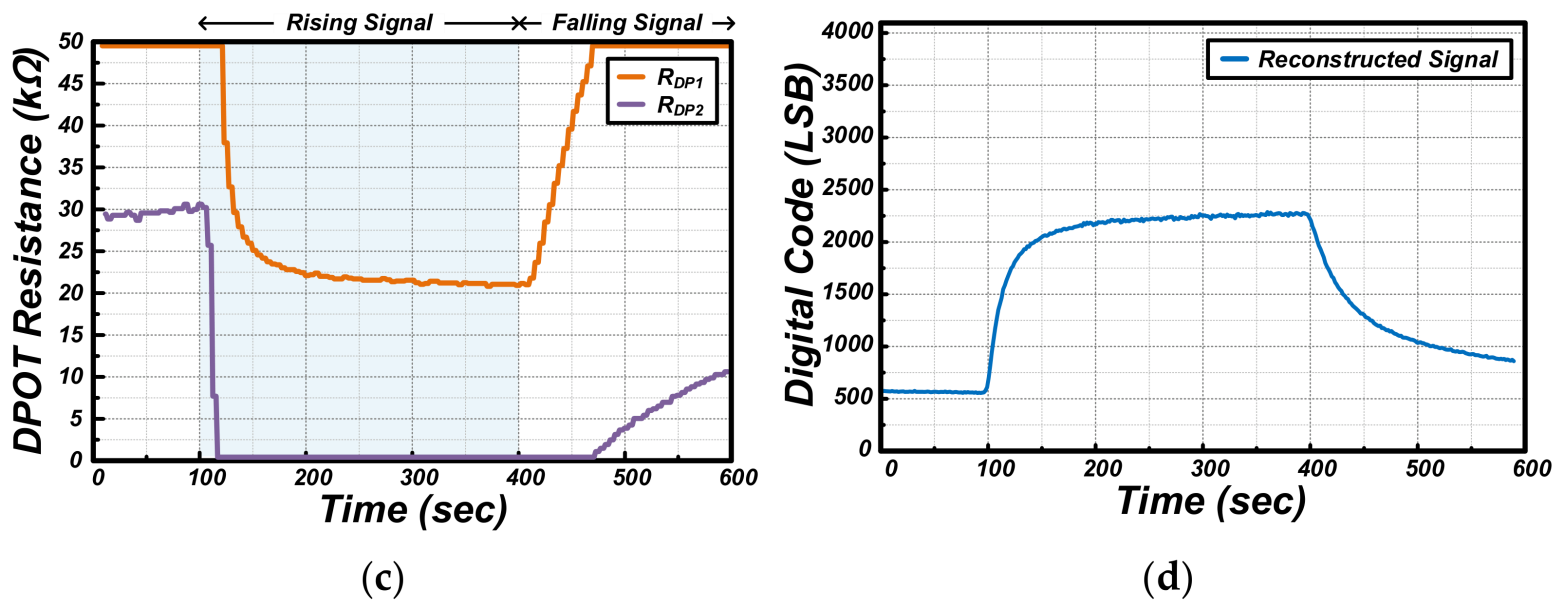
Measured waveforms of (**a**) V_Buf_, (**b**) VADC, and (**c**) DPOT resistance during ethanol exposure, with (**d**) the reconstructed digital signal.

The real-time operational characteristics of the proposed adaptive interface were analyzed by exposing the TGS-2600 sensor to ethanol gas (20 ppm, 500 sccm flow rate) under harsh environmental conditions (25 °C, 80% RH). Figure 7 illustrates the voltage waveforms at each circuit node, the corresponding resistance variations in the digital potentiometers, and the final reconstructed data over time, both before and after gas exposure. As shown in the V_Buf_ waveform in Figure 7a, the voltage rises sharply following the injection of ethanol gas at approximately 100 s due to the rapid decrease in sensor resistance. This signal, which already started from a high baseline due to the high-humidity environment, surged immediately after injection to a peak of approximately 2.75 V, well beyond the 1.2 V physical input limit of the ADC. In a conventional AFE using a fixed-gain method, signal saturation would occur in this region, resulting in the complete loss of gas concentration information.

However, the proposed adaptive algorithm performs real-time control to maintain the ADC input voltage (V_ADC_) within the effective range (0.3 V–0.9 V) around the V_CM_ of 0.6 V, as shown in Figure 7b. This is achieved through the dynamic changes in the DPOT resistances (R_DP1_, R_DP2_) illustrated in Figure 7c. At the moment V_Buf_ rises, the MCU instantaneously calculates and updates the attenuation ratio and gain to bring the signal back to V_CM_ at the next sampling point. Consequently, the V_ADC_ waveform is maintained within a stable operating range, exhibiting a fine sawtooth pattern without significant fluctuations.

Finally, the results reconstructed by combining the ADC measurements with the DPOT resistance variations updated in each cycle within the MCU are shown in Figure 7d. The Reconstructed Digital Signal successfully restores the data across the entire range that would have been clipped due to input limits, accurately following the variation patterns of the original V_Buf_ signal in LSB-scale digital codes. This demonstrates that the proposed interface has successfully extended the physical hardware dynamic range through a software-defined approach.

**Figure 8:**
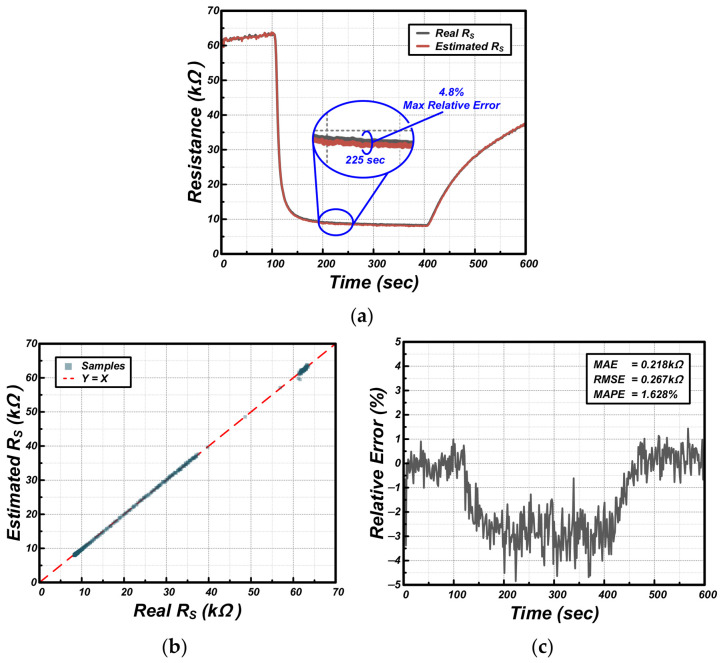
Accuracy results: (**a**) RS comparison, (**b**) linearity, and (**c**) relative error.

Based on the V_ADC_ data acquired through the adaptive interface and the real-time recorded DPOT codes, we performed a comparative analysis between the Estimated Sensor Resistance (Estimated R_S_) inversely calculated by the MCU and the Actual Sensor Resistance (Real R_S_). Figure 8 presents the resistance reconstruction results and the corresponding statistical error metrics across the entire ethanol gas exposure experiment.

As shown in Figure 8a, the estimated R_S_ precisely follows the actual sensor resistance throughout the rapid gas response at 100 s and the subsequent recovery phase. Although V_ADC_ was maintained as a sawtooth waveform near 0.6 V, as seen in Figure 7b, the data in the physical saturation region (the data loss region in conventional methods) was perfectly reconstructed by inversely applying the updated attenuation and amplification gains to the transfer function. The Maximum Relative Error during the experiment was approximately 4.8%, which occurred around 225 s when the gas concentration was at its peak and sensor resistance was at its lowest.

To quantitatively evaluate the linearity and accuracy of the reconstructed data, a scatter plot analysis was conducted, as shown in Figure 8b. The results show that the estimated and actual values are almost perfectly aligned with the Y = X line. Furthermore, the time-series relative error graph in Figure 8c confirms excellent performance metrics, with a Mean Absolute Error (MAE) of 0.218 kΩ and a Mean Absolute Percentage Error (MAPE) of 1.628% across the entire experimental period. In particular, Figure 8 presents a transient comparison between the original and reconstructed signals on a time axis, illustrating the high fidelity of the signal recovery even during rapid hardware parameter transitions. Through more than five independent experimental repetitions, it was confirmed that the reconstruction error remains consistently within the range shown in Figure 8 across all transient phases. This demonstrates that the real-time signal reconstruction capability of the proposed algorithm is not only precise but also statistically stable and highly repeatable, ensuring reliable data acquisition under continuous environmental fluctuations. These results prove that the proposed adaptive interface does not merely prevent signal saturation; it maintains a high level of data integrity by instantaneously reflecting variable hardware parameters (DPOT resistance values) into the calculation process. Furthermore, the proposed AFE ensures robust anti-interference performance by treating complex environmental fluctuations as baseline offsets and actively neutralizing them through real-time V_cm_ tracking and adaptive gain control. This hardware-level adaptation prevents signal saturation and maintains high signal integrity even under unpredictable industrial noise.

To evaluate the objective performance of the proposed adaptive gas sensor interface, its key specifications are compared with several recently reported systems: a self-powered wearable gas sensing system [26], an IoT infrastructure for indoor monitoring [27], and state-of-the-art platforms from 2025 including a power-efficient flue gas monitoring system [28] and an adaptive ROIC-based system for biomedical applications [29], as summarized in Table 1. The comparative analysis reveals that the present work demonstrates remarkable efficiency in terms of system resources and power consumption. While the reference systems utilize high-performance 32-bit MCUs or energy-intensive processing units such as the CC2640R2F (27 mW) in [26] and the BCM2835 (700 mW) in [27], this work achieves ultra-low power operation at just 2.88 mW by adopting an 8-bit MCU (STM8L151C3). This represents a power reduction of approximately 9 to 240 times compared to the reference studies, maintaining a competitive edge even against the most recent low-power architectures [28,29].

The originality of this research is particularly evident in the design approach of the ADC interface. Whereas contemporary methods [26,27,28,29] often attempt to physically resolve signal saturation by securing wide ADC input ranges (e.g., 3.0 V or 5.0 V), the ADC used in this study successfully processes a wide range of input signals exceeding 2.75 V despite its extremely limited 1.2 V input range. This is achieved through the real-time V_CM_ tracking algorithm, which logically overcomes physical hardware constraints through intelligent control, a stark contrast to methods that rely heavily on hardware specifications to secure dynamic range. By employing optimized adaptive control logic instead of expensive high-performance processors, this study confirms that high-precision gas sensing is feasible even in low-specification, low-power environments. These characteristics suggest that the proposed interface is an ideal solution as a versatile hardware framework for compact IoT devices or industrial gas monitoring environments requiring long-term independent operation.

## 5. Conclusions

This study presented a digital potentiometer-based adaptive gas sensor interface that effectively prevents signal saturation via real-time gain control. The proposed AFE maintains high data integrity across diverse sensing environments, regardless of extreme environmental fluctuations, complex gas mixture compositions, or varying load magnitudes. By logically extending the physically limited ADC dynamic range, this approach provides a reliable foundation for stable monitoring under unpredictable conditions. Moving forward, the proposed interface is expected to serve as a versatile hardware framework for next-generation multi-functional sensing systems. By ensuring the acquisition of intact data without signal loss, the system enables the clear extraction of characteristic features, such as transient response slopes and recovery profiles, which are essential for precise gas identification. Future research will leverage these refined features alongside pattern recognition techniques to address gas selectivity issues and significantly improve substance identification accuracy in advanced IoT environmental monitoring.

## Figures and Tables

**Table 1:** Performance summary and comparison.

Index	Ref.	This Work	Ref. 1 [26]	Ref. 2 [27]	Ref. 3 [28]	Ref. 4 [29]
**MCU**	**Product Name**	STM8L151C3	CC2640R2F	BCM2835	STM32L496G	ESP32-H2FH2
	**Word Size**	8 bits	32 bits	32 bits	32 bits	32 bits
	**Core Speed**	16 MHz	48 MHz	1 GHz	80 MHz	96 MHz
	**Power Consumption**	2.88 mW	27 mW	700 mW	625 mW	85.8 mW
**ADC**	**Product Name**	ADS7866	Internal ADC	MCP3008	Internal ADC	ADS7866
	**Resolution**	12-bit	12-bit	10-bit	12-bit	12-bit
	**Supply Voltage**	1.2 V	1.8 V–3.3 V	5 V	3.3 V	1.2 V
	**Input Range**	1.2 V	3.0 V	5 V	3.3 V	1.2 V
	**Power Consumption**	0.22 mW	2.475 mW	2.750 mW	2.739 mW	0.22 mW

## Data Availability

The original contributions presented in this study are included in the article. Further inquiries can be directed to the corresponding author.

## References

[1] Li Z., Yu J., Dong D., Yao G., Wei G., He A., Wu H., Zhu H., Huang Z., Tang Z. (2023). E-nose based on a high-integrated and low-power metal oxide gas sensor array. Sens. Actuator B Chem..

[2] Wang L., Cheng Y., Gopalan S., Luo F., Amreen K., Singh R.K., Goel S., Lin Z., Naidu R. (2023). Review and Perspective: Gas Separation and Discrimination Technologies for Current Gas Sensors in Environmental Applications. ACS Sens..

[3] Chesler P., Hornoiu C. (2023). MOX-Based Resistive Gas Sensors with Different Types of Sensitive Materials (Powders, Pellets, Films), Used in Environmental Chemistry. Chemosensors.

[4] Khorramifar A., Karami H., Lvova L., Kolouri A., Łazuka E., Piłat-Rożek M., Łagód G., Ramos J., Lozano J., Kaveh M. (2023). Environmental Engineering Applications of Electronic Nose Systems Based on MOX Gas Sensors. Sensors.

[5] Se H., Song K., Liu H., Zhang W., Wang X., Liu J. (2023). A dual drift compensation framework based on subspace learning and cross-domain adaptive extreme learning machine for gas sensors. Knowl.-Based Syst..

[6] Liang Y., Wang J., Tian F., Su J. (2023). A novel temperature compensation approach of IR gas sensors in coal mines. Fuel.

[7] Sun Y., Zheng Y. (2023). A method of gas sensor drift compensation based on intrinsic characteristics of response curve. Sci. Rep..

[8] Lee D.-Y., Yu J.-B., Byun H.-G., Kim H.-J. (2022). Chemoresistive Sensor Readout Circuit Design for Detecting Gases with Slow Response Time Characteristics. Sensors.

[9] Ziyatdinov A., Marco S., Chaudry A., Persaud K., Caminal P., Perera A. (2010). Drift compensation of gas sensor array data by common principal component analysis. Sens. Actuator B Chem..

[10] Abdullah A.N., Kamarudin K., Kamarudin L.M., Adom A.H., Mamduh S.M., Mohd Juffry Z.H., Bennetts V.H. (2022). Correction Model for Metal Oxide Sensor Drift Caused by Ambient Temperature and Humidity. Sensors.

[11] Singh A., Sikarwar S., Verma A., Chandra Yadav B. (2021). The recent development of metal oxide heterostructures based gas sensor, their future opportunities and challenges: A review. Sens. Actuat. A-Phys..

[12] Liu H., Zhang L., Li K.H., Tan O.K. (2018). Microhotplates for Metal Oxide Semiconductor Gas Sensor Applications—Towards the CMOS-MEMS Monolithic Approach. Micromachines.

[13] Chang I.S., Byun S.W., Lim T.B., Park G.M. (2023). A Study of Drift Effect in a Popular Metal Oxide Sensor and Gas Recognition Using Public Gas Datasets. IEEE Access.

[14] Ren M., Xu H., Dong C., Zhang Z. (2022). Toward a Gas Sensor Interface Circuit—A Review. IEEE Sens. J..

[15] Kwon S.K., Byun H.G., Kim H.J. (2024). An Analog Adaptive Reference Generation Readout Integrated Circuit for Baseline-Free Gas Sensor Measurements. IEEE Trans. Instrum. Meas..

[16] Choi S., Park C.S., Chae H.Y., Oh B., Lee J., Kwon Y.M., Baik J.M., Shin H., Kim J.J. (2021). A Wide Dynamic Range Multi-Sensor ROIC for Portable Environmental Monitoring Systems with Two-Step Self-Optimization Schemes. IEEE Trans. Circuits Syst. I Regul. Pap..

[17] Grassi M., Malcovati P., Baschirotto A. (2007). A 160 dB Equivalent Dynamic Range Auto-Scaling Interface for Resistive Gas Sensors Arrays. IEEE J. Solid-State Circuits.

[18] Park K., Choi S., Chae H.Y., Park C.S., Lee S., Lim Y., Shin H., Kim J.J. (2020). An Energy-Efficient Multimode Multichannel Gas-Sensor System with Learning-Based Optimization and Self-Calibration Schemes. IEEE Trans. Ind. Electron..

[19] Hammer C., Warmer J., Maurer S., Kaul P., Thoelen R., Jung N. (2020). A Compact 16 Channel Embedded System with High Dynamic Range Readout and Heater Management for Semiconducting Metal Oxide Gas Sensors. Electronics.

[20] Gao M., Zhang B., Du X., Yang X., Zhai C., Chen C., Zhang Z., Zhao W. (2025). InSe-Based Sensors and Related Sensing Systems for NO2 Gas/Temperature Monitoring. ACS Sens..

[21] Figaro USA Inc TGS 2600 PRODUCT INFORMATION. https://www.figarosensor.com/product/docs/TGS2600B00%20(0913).pdf.

[22] Chai H., Zheng Z., Liu K., Xu J., Wu K., Luo Y., Liao H., Debliquy M., Zhang C. (2022). Stability of Metal Oxide Semiconductor Gas Sensors: A Review. IEEE Sens. J..

[23] Kim K., Park J.K., Lee J., Kwon Y.J., Choi H., Yang S.-M., Lee J.-H., Jeong Y.K. (2022). Synergistic approach to simultaneously improve response and humidity-independence of metal-oxide gas sensors. J. Hazard. Mater..

[24] STMicroelectronics STM8L151C3 Datasheet. https://www.st.com/resource/en/datasheet/stm8l151c4.pdf.

[25] Texas Instruments Incorporated, ADS7866 Datasheet. https://www.ti.com/lit/ds/symlink/ads7867.pdf?ts=1774852639314&ref_url=https%253A%252F%252Fwww.google.com%252F.

[26] Wu Z., Wang H., Ding Q., Tao K., Shi W., Liu C., Chen J., Wu J. (2023). A Self-Powered, Rechargeable, and Wearable Hydrogel Patch for Wireless Gas Detection with Extraordinary Performance. Adv. Funct. Mater..

[27] Anik S.M.H., Gao X., Meng N., Agee P.R., McCoy A.P. (2022). A cost-effective, scalable, and portable IoT data infrastructure for indoor environment sensing. J. Build. Eng..

[28] Shukla P., Goel A., Kumar V., Kaur H., Malav V.K., Rawat B. (2025). A Portable and Power-Efficient Flue Gas Monitoring System for Real-Time Air Quality Measurement. IEEE Sens. J..

[29] Kim J.-N., Kwon S.-K., Park B.-C., Kim H.-J. (2025). A Low-Power Portable Gas Sensor System with Adaptive ROIC and Wi-Fi Communication for Biomedical Applications. Chemosensors.

